# Cognitive performance, sleep quality and physical activity as predictors of functional mobility in older adults

**DOI:** 10.3389/fnagi.2025.1649682

**Published:** 2025-09-10

**Authors:** Daniela Smirni, Garden Tabacchi, Roberta Cottone, Giovanni Angelo Navarra, Giulio Muscarnera, Vincenzo Di Noto, Antonino Scardina, Marianna Bellafiore

**Affiliations:** ^1^Department of Psychology, Educational Science and Human Movement, University of Palermo, Palermo, Italy; ^2^Department of Neuroscience, Biomedicine and Movement, University of Verona, Verona, Italy; ^3^Azienda Sanitaria Provinciale di Palermo, Palermo, Italy

**Keywords:** executive functions, visuomotor coordination, grooved pegboard test, timed up and go test, dynamic balance, fall risk

## Abstract

**Objective:**

This study investigated the relationships between functional mobility and cognitive performance, sleep quality and physical activity in older adults according to age, sex, weight status and education, and whether these variables could be predictors of functional mobility and fall risk.

**Method:**

Eighty-five physically independent older adults (72.3 ± 5.67 years; 83.5% female), without significant cognitive impairments, were involved in this cross-sectional study. Functional mobility, cognitive functions as phonemic fluency, attention, memory, processing speed, and manual dexterity were, respectively, assessed with Timed Up and Go (TUG) test, phonemic fluency test, Grooved Pegboard Test, (GPT) and Symbol Digit Modalities Test, Stroop Color-Word Test. Sleep quality and physical activity were evaluated with self-reported questionnaires. Correlational and multiple regression analyses were performed.

**Results:**

Poorer TUG performance was significantly associated with older age (*ρ* = 0.46, *p* < 0.001), lower educational level, reduced GPT performance, and lower scores on working memory, and processing speed. Age and GPT performance showed the strongest associations with TUG results. Regression analysis confirmed age and manual dexterity as significant predictors of functional mobility.

**Conclusion:**

Older age and reduced manual dexterity were linked to greater fall risk. These findings suggest that early interventions targeting fine motor skills may help preserve mobility and prevent falls in aging populations.

## Introduction

According to estimates from The World Health Organization, by 2050 the number of people aged 60 and over will double worldwide, while the number of people aged 80 and over will triple by the same date, reaching 426 million (World Health Organization [WHO], 2022).

These demographic trend estimates will inevitably be associated with a significantly more extensive healthcare cost for the health needs of this people. The prevention of health and the well-being of an ever-growing population, therefore, must be considered as a priority health area. Even more so because aging can have a significant impact on people’s cognitive, emotional, social and physical well-being and on their ability to act autonomously and independently in the complex or even ordinary tasks of daily life (Fulop et al., 2019; Navarra et al., 2023).

Motor difficulties, especially those related to balance (Gamwell et al., 2022; Larsson et al., 2019) and falls (Pirker and Katzenschlager, 2017; Appeadu and Bordoni, 2023), which often lead to hospitalizations, are common among the elderly population. The WHO even estimates that deaths from falls are more frequent than those from any other injury (World Health Organization [WHO], 2021).

Among the risk factors for falls, the literature includes possible cognitive difficulties (Van Doorn et al., 2003). Aging is often associated with a gradual impairment of cognitive functions, especially the prefrontal more integrated higher-order processes (Harada et al., 2013; Smirni et al., 2019; Borghesani et al., 2013; Gatica et al., 2021; Wilson et al., 2007; Stolwyk et al., 2015).

Sleep deprivation, poor quality, and sleep disturbance, such as insomnia, and sleep-related breathing disorders (Dzierzewski et al., 2017; Guarnieri and Sorbi, 2015; Wang et al., 2024), can promote cognitive decline and have destabilizing effects on postural control and the risk of falls in older adults (Izadi et al., 2022; Robillard et al., 2011). Conversely, good sleep quality promotes better brain function, particularly in working memory, switching ability, verbal fluency and recall (Wilckens et al., 2018).

Similarly, several studies have explored the relationship between physical activity and cognition in the aging process (Giustino et al., 2023). Scherder’s review, for example, showed a positive correlation between cognitive performance and qualitative elements of physical activity, and documented that decline in functional mobility corresponds to a decline in cognitive function (Scherder et al., 2014). Therefore, in the study of the motor behavior of the elderly, not only the physical component but also the cognitive component of the movement should be considered. Especially since the literature has abundantly documented the relevance of physical activity on brain plasticity, cognitive function, and overall well-being (Ludyga et al., 2023; Mandolesi et al., 2018, 2024). Physical activity can decrease the likelihood of neurological disorders and shielding the brain from the adverse consequences of aging (Passarello et al., 2022; MacPherson et al., 2017). Observational studies showed that sustained physical exercise programs, characterized by moderate to vigorous intensity, improve memory, attentional processes, and increase executive functioning in middle-aged and elderly subjects (Gelfo et al., 2018; Mandolesi et al., 2018; Serra et al., 2022).

The integrity of the executive functions is fundamental in the planning and organization of any finalized activity and plays a key role in guiding appropriate behavior across various situations (Zheng et al., 2022). In older adults, executive functions are especially relevant for managing complex physical activities, for the regulation of gait and balance and for increases in balance and gait speed (van Iersel et al., 2008; Ödemişlioğlu-Aydın and Aksoy, 2023) and they can compensate for age-related declines in automatic motor control (Fastame et al., 2022).

While the association between executive functions and the Timed Up and Go (TUG) test is well documented (e.g., van Iersel et al., 2008; Fastame et al., 2022), the present study aims to advance this literature by adopting a more integrative and multidimensional approach. In addition to executive functions, we consider a broader spectrum of cognitive abilities, including verbal fluency, attention, processing speed, and various memory components, as well as sleep quality, physical activity levels, and demographic characteristics (age, sex, weight status, education). By examining the interplay among these variables, we aim to better understand their joint and individual contributions to functional mobility and fall risk. This comprehensive framework may offer deeper insight into the multifactorial nature of mobility outcomes in older adults and support the development of more targeted and effective interventions.

## Methods

### Study design and sample

The present study constitutes an observational investigation in which the relationship between functional mobility and cognitive performance, sleep quality and physical activity were examined. It is part of a project developed by the University of Palermo and the Provincial Health Authority of Palermo, called Promotion of Physical Activity and Prevention of Domestic Accidents (PAP and DAP) (Navarra et al., 2023). Eighty-five physically independent individuals (71 women, 83.5%), over the age of 60, without physical limitations and cognitive impairments, from different neighborhoods of Palermo and its province, were recruited. The inclusion criteria ensured that only older adults without known cognitive or neurological diagnoses participated in the study. Specifically, the absence of cognitive and physical impairments was assessed through a combination of self-reported medical history and functional autonomy in daily life. Participants were recruited by Provincial Health Authority of Palermo, and were required to be independent in daily activities, free from overt motor or sensory deficits that could interfere with testing, and not currently undergoing treatment for psychiatric or neurodegenerative conditions. Individuals reporting prior diagnoses of dementia, Parkinson’s disease, stroke, or other conditions known to affect cognition or mobility were excluded. Data collection was conducted in three sessions, each lasting approximately 2 h, for a total of 6 h and were collected by specialists in sport and exercise sciences and psychologists. Demographic data, including age and sex of participants, were obtained through an anamnestic questionnaire. Additionally, anthropometric measurements, specifically body weight and height, were recorded. Body mass index (BMI) was subsequently calculated using the standard formula: weight (kg) divided by height squared (m^2^).

### Functional mobility performance, sleep quality ad physical activities level

To assess functional mobility, dynamic balance, gait, and fall risk, Timed Up and Go Test (TUG) was used. The TUG belongs to Senior Fitness Test, a test battery of motor performance tests specifically designed for individuals aged 60 years and older (Beauchet et al., 2011; Herman et al., 2011; Kear et al., 2017). This test assesses the duration required for an individual to rise from a chair (seat height: 40 cm, no armrests, fixed back), walk a distance of 3 m with shoes on a firm and stable floor up to a cone marked the turning point, execute a 180-degree turn, return to the chair, and sit down (Browne and Nair, 2018). Participants were prompted by a go signal from the specialist in sport and exercise sciences who instructed them to use a comfortable and safe walking speed. I Timing begins at the verbal cue “go” and stops once the participant is seated again. A longer completion time indicates poorer performance. Each participant performed the TUG test once; if a clear procedural error occurred, the test was repeated. The normative data show a worsening in performance with age: 8.1 s for the 60–69 s, 9.2 s for the 70–79 s, and 11.3 s for the 80–89 s (Bohannon, 2006).

The Physical activity (PA) status, categorized as inactive, sufficiently active, or very active, was assessed using the International Physical Activity Questionnaire for Italian Elderly (IPAQ-EIT). Moreover, this questionnaire was employed to gather data on participants’ level of education. This instrument consists of seven items designed to estimate the amount of physical activity performed over the previous 7 days. Participants reported the number of days and minutes spent engaging in physical activities of varying intensities, as well as the time spent walking and sitting during the past week. From these responses, a Metabolic Equivalent of Task (MET) score in minutes per week (MET min/week) was calculated. One MET represents the energy expenditure of an individual at rest. For each category of physical activity (walking, moderate, and vigorous), MET values were computed by multiplying the number of minutes spent on the activity, the number of days it was performed, and a standard MET coefficient assigned to that activity type (Abate Daga et al., 2021; Bertocchi et al., 2021). The total MET score was derived by summing the MET values for all activity types. Based on the total MET score, participants were classified as: inactive: <700 MET min/week; sufficiently active: 700–2,519 MET min/week; very active: ≥2,520 MET min/week.

The Sleep quality was assessed using the Pittsburgh Sleep Quality Index (PSQI), a standardized rating scale designed to evaluate overall sleep quality and its patterns over the past month (Buysse et al., 1989). The PSQI comprises 19 self-rated items, which are grouped into seven components: subjective sleep quality, sleep latency, sleep duration, habitual sleep efficiency, sleep disturbances, use of sleep medication, and daytime dysfunction. Each component is scored based on individual items or the sum of multiple items, with higher scores indicating greater severity of sleep-related problems. The component scores are then summed to yield a global PSQI score, ranging from 0 to 21. A global score greater than 5 is indicative of poor sleep quality or the presence of sleep disturbances, whereas a score of 5 or lower reflects good sleep quality (Devoto et al., 2016).

### Cognitive assessment

The phonemic fluency test (Benton et al., 1994; Novelli et al., 1986) was used to assess language production, executive function, and cognitive flexibility. Participants were instructed to generate as many words as possible within 1 min that begin with a specific letter, while excluding proper nouns and words that differ only by their suffixes. The letters used in the task were ‘F’, ‘A’, and ‘S’. A score is given for each word produced that correctly adheres to the provided rules, with a total duration of 3 min (1 min per letter). A score below 17.35 is considered indicative of a pathological condition.

A modified version of the Grooved Pegboard Test (GPT) was used to assess manual dexterity, fine motor control, visuomotor coordination, motor planning and speed, as well as precision and coordination (Lafayette Instrument Company, 2002; Trites, 1977; Kløve, 1963). Participants were required to insert as many keyhole-shaped pegs as possible into slotted holes within 1 min. The slots are oriented in various directions, increasing the task’s complexity compared to standard pegboards and requiring enhanced visuomotor integration and executive control. This task demands not only precise motor execution but also real-time visuospatial processing and executive planning, thereby offering valuable insights into the functioning of sensorimotor pathways, cortical–subcortical networks, and frontal-executive systems. The test was administered in three consecutive one-minute trials, each performed with the participant’s preferred hand. The total score, with a maximum of 75, was calculated as the cumulative number of pegs successfully inserted across all three trials.

The Symbol Digit Modalities Test was used to assess processing speed, attention, and working memory (Nocentini et al., 2006; Smith, 1982; Wechsler, 2008). The test presents a key in which nine unique symbols are each paired with a digit from 1 to 9. Participants are required to rapidly write the digit corresponding to each symbol by referring to the key. The task is time-limited to 90 s, during which participants must complete as many correct symbol-digit pairings as possible. The total score is determined by the number of correct associations made within the time limit. A score below 34.20 is considered indicative of impaired performance.

The Digit Span forward and backward test (Monaco et al., 2013), was used to assess short-term verbal memory span and verbal working memory span. Participants were required to repeat sequences of digits that progressively increase in length, both in the same order (forward) and in reverse order (backward). The span score for each condition is defined as the longest sequence of digits correctly recalled following a single presentation. The normative cut-off scores are 4.26 for the forward span and 2.65 for the backward span.

The short version of the Stroop Color-Word Test was used to assess interference inhibition, a component of executive functioning (Caffarra et al., 2002). The test evaluates both the number of ink colors correctly identified out of 30 color words and the time required to complete the task. Performance is considered within the normal range if the number of errors does not exceed 4.24 and the completion time is below 36.92 s.

### Statistical analysis

Statistical analyses were conducted using STATA/MP version 12.1 (StataCorp LP, College Station, TX, United States). The Shapiro-Francia test was employed to evaluate the normality of the distribution of the examined variables (Mbah and Paothong, 2015). For quantitative variables exhibiting normal distribution, means and standard deviations (SDs) were calculated. Non-normally distributed variables were reported as medians and interquartile ranges (IQRs). Categorical variables were summarized using absolute frequencies and percentages.

Linear correlations between quantitative variables were examined, with the TUG test result designated as the dependent variable. Pearson’s (*r*) and Spearman’s (*ρ*) correlation coefficients were used to assess bivariate relationships. When the independent variable was qualitative, the Student’s T test was used to compare the mean differences when two categories were present; when the independent variable had more than two categories, the one-way analysis of variance was used, with the Fisher value indicating differences between the means in the subcategories. Variables demonstrating significant correlations with TUG performance were subsequently entered into a multiple linear regression model to identify independent predictors. Regression analysis was conducted both on untransformed and log transformed skewed variables, and results were presented as unstandardized regression coefficients (Coef.) with standard errors (SE), as well as standardized beta coefficients (*β*). Statistical significance was defined as a *p*-value < 0.05.

### Results

Normality of distribution was assessed for all variables. The following measures were normally distributed: age, education, height, phonemic fluency, Symbol Digit Modalities Test, and Digit Span Forward. In contrast, the distributions of weight, body mass index (BMI), TUG performance, metabolic equivalent of task (MET), Pittsburgh Sleep Quality Index (PSQI) score, total time on the Grooved Pegboard Test, Digit Span Backward, and Stroop test performance showed non-normal distributions.

The Table 1 shows participants demographic and anthropometric measurements. The sample was predominantly composed of women (83.5%) and individuals aged 60–74 years (63.5%). Most participants were classified as overweight and obese (58.7%), with a median BMI of 25.7 Kg/m^2^. Additionally, 60.5% of the sample reported a high level of PA. Education level was high-medium, with a median of 13 years of formal schooling.

**Table 1:** Participants socio-demographic and anthropometric data.

Variables	Mean	SD
Age (yrs)	72.3	5.67
Height (m)	1.6	0.07

Variables	Median	IQR
Weight (Kg)	66.0	13.00
BMI (Kg/m^2^)	25.7	4.38
Education (yrs)	13.0	5.00

Variables	*N*	%
Sex		
Male	14	16.5
Female	71	83.5
Total	85	
Age (yrs)		
60–74	54	63.5
>74	31	36.5
Weight status		
Normal	33	41.3
Overweight	37	46.2
Obese	10	12.5
PA level		
Very active	43	60.5
Sufficiently active	20	28.2
Inactive	8	11.3

SD, standard deviations; IQR, InterQuartile ranges; N, number; %, percentage; BMI, Body Mass Index; PA, physical activity.

The Table 2 shows the descriptive statistics of functional mobility, cognitive functions, sleep quality and physical activity level in the sample.

**Table 2:** Mean and standard deviation, and median and interquartile range of cognitive and motor performance measures in the sample.

Variables	Mean	SD
Phonemic fluency score	30	9.65
Symbol/digit score	26.9	11.73
Digit span forward score	5.3	1.01

Variables	Median	IQR
TUG performance (seconds)	8.2	1.34
Total MET score	3,240	5,524.50
Grooved Pegboard score	58	20
PSQI score	5	6
Digit span backward score	3	1
Stroop time (seconds)	29	22
Stroop errors (number)	2	4

SD, standard deviation; IQR, interquartile range; TUG, timed up and go; MET, metabolic equivalent of task; PSQI, Pittsburgh Sleep Quality Index.

Among cognitive functions, participants exhibited a mean phonemic fluency score of 30 (SD = 9.65), a mean Symbol-Digit Modalities Test score of 26.9 (SD = 11.73), and a mean Digit Span Forward score of 5.3 (SD = 1.01), indicating moderate variability in verbal fluency, processing speed, and attention span, respectively.

The median TUG performance time was 8.2 s (IQR = 1.34), corresponding to a normal range of functional mobility in the sample. The GPT yielded a median score of 58 pegs inserted (IQR = 20), reflecting adequate levels of manual dexterity, visuospatial processing, and executive functioning. The median total MET value was 3,240 (IQR = 5,524.5), reflecting a wide range of PA levels within the group.

Sleep quality, as measured by the PSQI, showed a median score of 5 (IQR = 6), hovering around the clinical threshold for poor sleep quality. The Digit Span Backward task yielded a median score of 3 (IQR = 1), indicating a limited working memory capacity. Stroop Test results showed a median completion time of 29 s (IQR = 22) and a median of 2 errors (IQR = 4), reflecting substantial variability in inhibitory control and cognitive flexibility.

The correlation analysis revealed that TUG performance was significantly positively correlated with age (*ρ* = 0.46, *p* < 0.001), indicating that performance declined with increasing age. Conversely, TUG performance was significantly negatively correlated with educational level, GPT performance, Stroop Test completion time and error rate, Digit Span Backward score, and Symbol-Digit Modalities Test score (Table 3). Among these, the strongest correlations were observed with age (*ρ* = 0.46) and the GPT (*ρ* = −0.43), suggesting that older age and poorer manual dexterity were most strongly related to poorer TUG performance.

**Table 3:** Spearman’s rho correlation coefficients between the Timed Up and Go performance and motor and neuropsychological variables.

Variables	TUG performance (seconds)	
	Spearman’s ρ	*p*-value
Age (yrs)	0.46	0.000
Education (yrs)	−0.22	0.047
Weight (Kg)	−0.05	0.648
Height (m)	−0.12	0.285
BMI (Kg/m^2^)	0.03	0.782
Total MET score	−0.01	0.920
Grooved Pegboard score	−0.43	0.000
Stroop time (seconds)	0.25	0.020
Stroop errors (number)	0.33	0.002
Phonemic fluency score	−0.15	0.178
PSQI score	−0.12	0.442
Digit span forward score	−0.04	0.704
Digit span backward score	−0.22	0.045
Symbol/digit score	−0.37	0.000

TUG, timed up and go; BMI, Body Mass Index; MET, metabolic equivalent of task; PSQI, Pittsburgh Sleep Quality Index.

No significant correlations were observed between TUG performance and body weight, height, BMI, total metabolic equivalents, Pittsburgh Sleep Quality Index score, Digit Span Forward score, or phonemic fluency score. Additionally, total MET and physical activity status (active vs. inactive) did not show significant associations with TUG performance.

Further correlational analyses revealed no significant associations between the PSQI total score and age (*ρ* = −0.05, *p* = 0.84), sex (*ρ* = 0.01, *p* = 0.96), or education (*ρ* = 0.11, *p* = 0.65). With the notable exception of BMI, which showed a strong negative correlation with the PSQI score (*ρ* = −0.70, *p* = 0.0005), and weight (*ρ* = −0.56, *p* = 0.0088), indicating that lower BMI and weight were associated with better self-reported sleep quality in this sample. Regarding cognitive measures, no significant correlations were found between the PSQI total score and verbal fluency (*ρ* = 0.04, *p* = 0.86), forward (*ρ* = −0.0014, *p* = 0.99) or backward digit span (*ρ* = 0.07, *p* = 0.77), symbol digit (*ρ* = −0.04, p = 0.86), or Stroop test time (*ρ* = 0.04, *p* = 0.86) or Stroop test errors (*ρ* = −0.10, *p* = 0.65). The only significant association emerged with the Grooved Pegboard total score (*ρ* = 0.5789, *p* = 0.0060) (see Supplementary Data).

Table 4 shows the mean TUG performance, standard error, and group comparisons based on sex, weight status, and physical activity level.

**Table 4:** Group differences in timed up and go performance by sex, age, weight status, and physical activity level.

Variables	TUG performance			
	Mean	SE	*t* or *F*	*p*-value
Sex			−0.147*	0.884
Male	8.34	0.452		
Female	8.40	0.157		
Age			−4.58*	< 0.001
60–74 years	7.87	0.128		
>74 years	9.28	0.281		
Weight status			0.33^	0.719
Normal	8.30	0.412		
Overweight	8.42	0.342		
Obese	8.71	0.214		
PA level			0.19^	0.824
Very active	8.57	0.251		
Sufficiently active	8.38	0.249		
Inactive	7.25	0.400		

TUG, timed up and go; SE, standard error; PA, physical activity.

*t, *t* values; ^, *F* values.

No significant differences in TUG performance were observed between males and females (*t* = 0.147, *p* = 0.884), with both groups displaying nearly identical mean times (Male: 8.34 s; Female: 8.40 s). An independent samples *t*-test revealed a significant difference in functional mobility between younger (60–74 years) and older (>74 years) adults, *t*_(83)_ = −4.58, *p* < 0.001. Comparisons across weight status categories (normal weight, overweight, and obese) did not yield statistically significant differences (*F* = 0.33, *p* = 0.719), although a trend toward longer TUG times was noted in the obese group (Obese: 8.71 s vs. Normal weight: 8.30 s).

Physical activity level was also not significantly associated with TUG performance (*F* = 0.19, *p* = 0.824). While the mean TUG time appeared lower in the inactive group (7.25 s) compared to the very active (8.57 s) and sufficiently active (8.38 s) groups, this difference was not statistically significant, and may reflect variability due to the small subgroup size.

The multiple regression analysis identified age and the GPT performance as significant predictors of TUG performance (Table 5). In contrast, other cognitive variables, symbol digit modalities, digit span backward, Stroop time, and Stroop error, did not significantly predict TUG performance (*p* > 0.05). The overall model was significant, *R*^2^adj = 0.319, *F*(6, 78) = 7.56, *p* = 0.000. There was no evidence of multicollinearity, with the Variance Inflation Factor (VIF) < 5 for all the variables. There were no substantive differences in the results obtained on the log transformed variables compared to untransformed (see Supplementary Data).

**Table 5:** Results of multiple regression analysis predicting TUG performance.

Variables	TUG performance (seconds)				
	Coef.	SE	*t*	*p*-value	*β* coef.
Age (yrs)	0.09	0.027	3.17	0.002	0.35
Grooved Pegboard score	−0.03	0.011	−2.70	0.009	−0.29
Symbol/digit score	−0.01	0.015	−0.89	0.377	−0.11
Digit span backward score	−0.06	0.170	−0.33	0.744	−0.03
Stroop time (seconds)	−0.01	0.009	−1.16	0.249	−0.14
Stroop errors (number)	0.04	0.033	1.20	0.233	0.12

Coef., unstandardized coefficient; SE, standard error; t, *t*-test; β, standardized (beta) coefficient.

*R*^2^adj, 0.319, *F*(6,78), 7.56, *p* = 0.000. There was no evidence of multicollinearity (variance inflation factor <5 for all the variables).

## Discussion

The aim of this study was to investigate the relationships between functional mobility and cognitive functions, sleep quality and physical activity according to age, sex, weight status and education. In addition, we examined whether these variables were predictors of functional mobility and fall risk. In this study, TUG test was used as provides useful information on the functional mobility, dynamic balance and fall risk in older adults (Shumway-Cook et al., 2000). Indeed, impaired walking agility is often associated with a deficit in motor control and impairment of cognitive and executive functions, factors that contribute significantly to the increased risk of falls in this population (Montero-Odasso et al., 2012; Yogev-Seligmann et al., 2008). In this study, participants showed an above-average performance in TUG test suggesting that they have a low risk of falls. This result could be explained with the presence in our sample of a high percentage of participants (almost 90%) with an active lifestyle and may depend by greater motivation of this group to participate in this study.

The data demonstrated a significant positive correlation between age and TUG test, indicating that performance time increases with advancing age. This finding reflects the age-related decline in motor abilities such as agility and speed. Aging is associated with a range of physiological and neurological changes that adversely affect movement efficiency, including reductions in muscle strength, deterioration of sensory functions, impaired coordination, decrease in dynamic balance, and slower reaction times (Wu R. et al., 2021). These factors collectively contribute to decreased postural stability and prolonged execution times in motor tasks. The present findings are consistent with existing literature, which has established a clear relationship between age and both TUG performance time and overall mobility quality in older adults (Bohannon, 2006). In particular, it has found that performance in dynamic balance deteriorates significantly from the age of 70, with this decline accelerating after the age of 85 (Wu H. et al., 2021; Marchesi et al., 2022). Thus, age emerges as a critical determinant in the decline of functional mobility. Prolonged TUG times may serve as a practical and informative tool for evaluating mobility status and monitoring the progression of disability risk in older adults.

Sex-related differences have been shown to influence balance and functional mobility, with some evidence indicating that men may retain superior dynamic balance abilities compared to women, particularly in older age groups (Wu R. et al., 2021). However, in the present study, no significant sex differences were observed.

Regarding weight status, Gao et al. (2019) found that being overweight is associated with reduced speed and acceleration of the center of mass during walking, resulting in impaired dynamic balance and functional mobility. However, in our study no significant correlation was detected between functional mobility performance and weight status. The anthropometric analysis of our sample revealed that fewer than half of the participants were within the normal weight range (41.3%), and the remaining 58.7% classified as overweight and obese. This distribution is consistent with previous findings indicating a high prevalence of excess body weight among older adults. Age-related physiological changes, including reductions in basal metabolic rate, and progressive alterations in body composition, such as increased adiposity and loss of lean muscle mass, contribute to the accumulation of excess weight in later life (Stenholm et al., 2014; Zamboni et al., 2008).

Given that most of the study participants were physically active, a good sleep quality was expected. However, the mean score recorded (5.0) approaches the threshold for normal sleep quality. This apparent discrepancy may be explained by age-related physiological changes that typically affect sleep in older adults, such as increased nocturnal awakenings and greater sleep fragmentation, which collectively reduce sleep efficiency, particularly after the age of 60 (Ohayon et al., 2004). Additionally, alterations in circadian rhythms and a decline in melatonin secretion have been identified as further contributors to the deterioration of sleep quality in aging populations (Hood and Amir, 2017).

It is worth noting that the absence of significant differences related to sex, weight, and physical activity level in our study may be partly explained by characteristics of the sample itself. First, the relatively small sample size may have limited the statistical power to detect subtle group differences. More importantly, the homogeneity of the sample, particularly in terms of physical activity, may have reduced between-subject variability. As most participants reported engaging in regular physical activity, their relatively preserved functional status might have attenuated the impact of sex and weight-related differences typically observed in more heterogeneous or sedentary populations. This ceiling effect may have masked potential associations, suggesting the need for further studies with larger and more diverse samples to better capture the influence of these variables on functional mobility in older adults.

Furthermore, in older adults, sleep disturbances have been closely linked to an increased risk of falls, domestic accidents, fatigue, physical weakness, impaired postural stability, and reduced motor performance (Sagayadevan et al., 2017). Despite these factors, impaired sleep quality did not appear to negatively influence balance or functional mobility in the present study. The absence of significant associations between PSQI scores and most demographic variables (age, gender, and education) suggests that, within this sample, subjective sleep quality was not strongly influenced by these factors. The strong negative correlations with BMI and weight indicate that participants with lower body mass and weight tended to report better sleep quality. This finding aligns with previous literature linking higher BMI to poorer sleep, potentially due to mechanisms such as increased risk of sleep-disordered breathing, systemic inflammation, or metabolic dysregulation (Chaput et al., 2012; Meyer et al., 2012).

In terms of cognitive functioning, the lack of correlations between PSQI scores and measures of verbal fluency, working memory (digit span), processing speed (symbol digit), and cognitive inhibition (Stroop) suggests that poorer subjective sleep quality in this group was not broadly associated with deficits in these domains. The sole significant association with Grooved Pegboard performance may point to a more specific link between sleep quality and sensorimotor functioning. This is consistent with evidence that sleep loss or fragmentation can impair motor coordination and increase reaction times (Alhola and Polo-Kantola, 2007), possibly reflecting underlying neural mechanisms distinct from those affecting higher-order cognition.

Interestingly, an inverse correlation was observed between educational level and TUG performance, suggesting that individuals with higher education exhibited better mobility and agility. In line with these findings, the study conducted by Hoogendijk et al. (2014), demonstrated a significant association between low education level and a higher prevalence of frailty, identifying education as a relevant determinant of functional health in older adults.

Several studies have reported that older adults who maintain an active lifestyle demonstrate superior performance in body balance assessments compared to their sedentary counterparts, suggesting that regular physical activity may help mitigate age-related declines in balance (Sarto et al., 2022; Papalia et al., 2020). In our study, data analysis showed a high level of physical activity and elevated average energy expenditure, expressed in MET, within the sample. The total MET score accurately reflects the intensity, duration, and frequency of physical activity, as suggested by Iona et al. (2022). In this context, the high energy expenditure observed indicates not only a good level of habitual physical activity but also a generally healthy lifestyle associated with greater functional autonomy. Importantly, stratification by physical activity levels did not yield any statistically significant differences in test performance. In contrast, significant associations were found between performance on the TUG test and several neuropsychological measures. Indeed, performance of GPT, Stroop Test (in terms of both completion time and number of errors), Digit Span Backward task, and Symbol-Digit Modalities Test were significantly correlated with TUG performance. Participants with better times in TUG performance demonstrated better outcomes on tasks assessing complex attention and visuomotor coordination. In particular, GPT is a well-established neuropsychological measure commonly used to assess fine motor coordination, manual dexterity, and psychomotor speed (Yao et al., 2020). Several studies have suggested that GPT performance involves attentional processes, motor planning, and speed–accuracy trade-offs, and may serve as a more sensitive indicator of cognitive functioning than of pure motor performance (Ashendorf et al., 2009; Bezdicek et al., 2014; Tolle et al., 2020). A reduction in hand motor function has been associated with a broader deterioration in executive functioning (Kobayashi-Cuya et al., 2018; Stuhr et al., 2018). Additionally, research suggests that GPT is useful in detecting subtle motor slowing or coordination deficits in clinical conditions such as traumatic brain injury, multiple sclerosis, Parkinson’s disease, and dementia.

Similarly, the Stroop Test requires the ability to selectively maintain attention on a specific aspect of a stimulus while inhibiting interference from irrelevant information over sustained periods. Prolonged response times or a high number of errors indicate difficulties in interference control and cognitive inhibition. Likewise, the Digit Span Backward task involves the retention of a sequence of digits in the correct forward order, followed by its active manipulation in reverse order. This task places demands on complex attentional processes and working memory, and it is known to engage frontal and prefrontal brain regions (Bahri et al., 2024; Hale et al., 2002). Similarly, the Symbol-Digit Modalities Test involves rapid symbol substitution within a limited time frame, relying on processing speed, learning, and working memory.

In our study, the significant correlation between TUG performance and processing speed, sustained and selective attention, interference inhibition, attention maintenance, and working memory, suggests that these cognitive-executive functions may affects the functional mobility and fall risk in older adults. Specifically, TUG task requires sustained and selective attention to remain focused on the motor sequence (e.g., standing up, walking, turning) while ignoring potential environmental distractions. It also engages executive functions involved in planning the sequence of actions (‘stand up, walk, turn, return, sit down’), inhibition to prevent premature or inappropriate movements, cognitive flexibility to adapt to unexpected obstacles or difficulties, and working memory to retain the steps of the sequence during execution. These cognitive demands implicate the involvement of prefrontal brain regions, like those activated in tasks such as the Digit Span, Stroop, Symbol-Digit Modalities, and Grooved Pegboard Tests.

In our study, in line with the results showing the strongest associations of age and GPT with TUG performance, the regression analysis confirmed the age and manual dexterity as significant predictors of functional mobility. Although TUG and GPT assess distinct motor domains, gross motor coordination and fine motor dexterity, respectively, their shared neuromotor underpinnings likely account for the observed relationship between manual dexterity and functional mobility (Yao et al., 2020). However, given the correlational design of the present study, no causal conclusions can be drawn, and further longitudinal or interventional research is needed to explore the potential interplay between fine and gross motor abilities in aging. Both tasks involve cortical motor areas, the cerebellum, and sensorimotor integration processes (Proville et al., 2014). In particular, the execution of each task relies on the planning and regulation of movement, proprioceptive feedback, and cognitive control. Despite targeting different effectors, these overlapping neural substrates suggest a degree of functional interdependence between fine and gross motor control (Rostami et al., 2025).

The relevance of functional mobility and their potential cognitive correlates, as highlighted in this study, supports our working hypothesis and aligns with existing literature suggesting that functional mobility and fall risk may have a significant cognitive component.

These findings have important clinical and preventive implications. Given the close relationship between cognitive-executive functions and TUG performance, incorporating cognitive assessments alongside motor evaluations (e.g., combining the TUG with brief tests of attention, inhibition, or working memory) may enhance early detection of older adults at increased risk of mobility decline and falls. This integrated approach could support more comprehensive screening protocols in clinical and community settings, allowing for timely and personalized interventions.

Furthermore, our results highlight the importance of considering individual factors such as education level and manual dexterity in the development of fall prevention strategies. Lower educational attainment may be associated with reduced cognitive reserve, potentially exacerbating the impact of executive dysfunctions on mobility. Similarly, reduced manual dexterity, often linked to poorer performance in cognitive-motor integration tasks, may serve as an early marker of functional decline. Tailoring prevention programs to these individual characteristics may improve their efficacy and support healthy aging trajectories.

### Limitations

Some limitations should be considered when interpreting the findings of this study. First, although participants were selected based on the absence of diagnosed neurological or psychiatric conditions and reported no history of cognitive decline, the lack of a standardized cognitive screening tool for their recruitment limits the validity of the conclusions regarding their cognitive integrity. Furthermore, data on history of falls and fear of falling, two factors closely related to functional mobility, were not collected. Including these measures would have provided valuable context for interpreting the TUG performance and its relationship with fall risk. Future studies should incorporate both cognitive screening tools and validated assessments of fall history and fall-related self-efficacy to strengthen the clinical relevance and ecological validity of the findings.

In addition, the sample was predominantly composed of female participants and individuals with relatively high educational attainment, which may limit the generalizability of the results to the broader older adult population, particularly males and those with lower educational levels (Salthouse, 2010; Stern, 2009). Additionally, the age distribution was skewed toward younger older adults (60–74 years), underrepresenting individuals aged 75 and above who may exhibit different cognitive and motor trajectories (Verghese, 2006). Physical activity was assessed through self-report and classified into broad activity categories, which may be susceptible to recall bias (Prince et al., 2008). Similarly, the use of the Pittsburgh Sleep Quality Index (PSQI), which, although widely validated and commonly employed in aging research, relies on self-reported data. The lack of objective sleep assessments, such as actigraphy or polysomnography, limits the precision of the findings related to sleep. Future studies should consider incorporating both subjective and objective measures to obtain a more accurate and comprehensive evaluation of sleep quality in older adults. The cross-sectional design of the study also precludes causal inference. Finally, the use of a single functional mobility measure (TUG) may not fully capture the multifaceted nature of motor function in aging; future studies should incorporate more sensitive and objective assessments such as gait analysis or dual-task paradigms (Montero-Odasso et al., 2012).

## Conclusion

Our findings support the hypothesis that movement control and dynamic balance are significantly correlated with higher-order cognitive processes. These results suggest that falls in older adults may not solely be attributed to physical decline but may also reflect diminished cognitive control mechanisms. Among the variables examined, manual dexterity was found to significantly predict functional mobility performance, underscoring the contribution of fine motor control to overall functional capacity in aging individuals.

In conclusion, the study shows that individuals at greatest risk of falls are those of more advanced age and those exhibiting poorer performance in manual dexterity. Therefore, functional mobility and fall prevention may be improved in older adults implementing early interventions targeting manual dexterity.

## Data Availability

The raw data supporting the conclusions of this article will be made available by the authors, without undue reservation.
