# Supplementary material for: Cognitive performance, sleep quality and physical activity as predictors of functional mobility in older adults
Source: Front Aging Neurosci. 2025 Sep 10;17:1649682. doi: 10.3389/fnagi.2025.1649682 (PMC12457295; doi:10.3389/fnagi.2025.1649682)
Supplement: Supplementary file 1 [file Supplementary_file_1.docx]

*#1*

*Results of the multiple regression analysis (predictors: age and total Grooved), conducted on log-transformed variables due to right-skewed distributions; coefficients, standardized beta coefficients, and significance values are reported, showing comparable outcomes to analyses performed without logarithmic transformation*

***#1 caption:*** *Results of multiple regression analysis with predictors age and total groove on log-transformed variables (right-skewed distributions). Reported are coefficients, standardized beta coefficients, and significance values, showing outcomes comparable to analyses without logarithmic transformation*

*#2*

*Variance Inflation Factors (VIF) for the predictors included in the regression models, computed in STATA to assess multicollinearity. VIF values indicate how much each predictor’s variance is inflated by the presence of the other predictors in the model. As commonly reported in the literature, VIF values between 1 and 5 are generally acceptable, whereas values ≥5–10 suggest potential multicollinearity issues (Kutner et al., 2005; see also* [*https://sscc.wisc.edu/sscc/pubs/RegDiag-Stata/no-multicollinearity.html*](https://sscc.wisc.edu/sscc/pubs/RegDiag-Stata/no-multicollinearity.html?utm_source=chatgpt.com)*). Only the significance of individual predictors is reported in the manuscript, as multicollinearity was excluded based on these results*

***#2 caption:*** *Variance Inflation Factors (VIF) of the predictors included in the regression models, computed in STATA to assess multicollinearity (values between 1–5 acceptable; ≥5–10 indicating potential issues).*
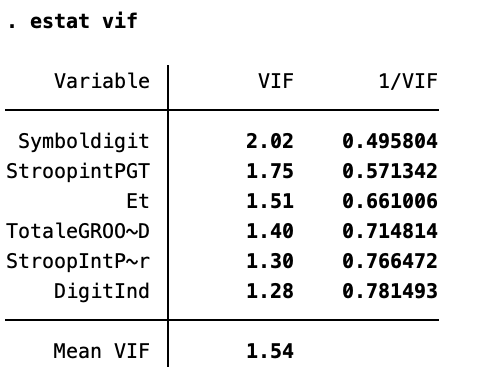


R2adj = 0.293, F(6, 78) = 6.79, p = 0.000. There was no evidence of multicollinearity (Variance Inflation Factor <5 for all the variables).

*#3*

*additional correlational analyses (supplementary data) to specifically examine the relationship between sleep quality (PSQI total score) and cognitive measures in the sample.*

***#3 caption:*** *Spearman’s rho correlation between the PSQI total score and demographic and cognitive variables*

|  | **PSQIScore** |  |  |
| --- | --- | --- | --- |
|  | correl | n.observations | *p-value* |
| **TUG** | -0.1855 | 21 | *0.4207* |
| **Sex** | 0.0113 | 21 | *0.9611* |
| **School years** | 0.1060 | 21 | *0.6475* |
| **Hand** | 0.0340 | 21 | *0.8836* |
| **Age** | -0.0470 | 21 | *0.8395* |
| **height** | -0.0214 | 21 | *0.9266* |
| **weight** | -0.5568 | 21 | *0.0088* |
| **BMI** | -0.6955 | 21 | *0.0005* |
| **Total MET** | 0.0161 | 21 | *0.9449* |
| **PA Very active** | 0.0241 | 21 | *0.9175* |
| **PA Sufficiently active** | -0.0270 | 21 | *0.9074* |
| **PA Inactive** | 0.0145 | 21 | *0.9503* |
| **Phonemic fluency** | 0.0420 | 21 | *0.8566* |
| **Total GROOVED** | 0.5789 | 21 | *0.0060* |
| **Symbol digit** | -0.0394 | 21 | *0.8654* |
| **Digit forward** | -0.0014 | 21 | *0.9953* |
| **Digit Back** | 0.0670 | 21 | *0.7729* |
| **Stroop Time** | 0.0390 | 21 | *0.8666* |
| **Stroop error** | -0.1041 | 21 | *0.6533* |
|  |  |  |  |
